# On the Purported Dichotomy Between Fake and Real Symptoms: The Case of Conversion Disorders

**DOI:** 10.3389/fpsyg.2019.02114

**Published:** 2019-09-18

**Authors:** Henrik Kessler, Nikolai Axmacher, Martin Diers, Stephan Herpertz

**Affiliations:** ^1^Department of Psychosomatic Medicine and Psychotherapy, LWL University Hospital, Ruhr University Bochum, Bochum, Germany; ^2^Department of Neuropsychology, Faculty of Psychology, Institute of Cognitive Neuroscience, Ruhr-University Bochum, Bochum, Germany

**Keywords:** functional neurological disorders, conversion, epistemology, hysteria, functional neuroimaging

## Introduction

Functional neurological disorders—classically labeled as “hysteria”—are among the most common conditions leading to admissions to neurological services. This term has been abundantly criticized for both methodological and ethical reasons. More recently, a clinical commentary that appeared in January 2019 (Madva et al., 2019) emphasized a specific aspect of this criticisms: The authors appeal to drop the term “hysteria” because evidence from functional neuroimaging shows that these symptoms have a clear neurobiological basis and are therefore not “faked.” They first cite functional neuroimaging studies that show distinctive brain activations in patients diagnosed with “conversion weakness” as compared to healthy subjects instructed to mimic a motor deficit. They conclude that “[…] these findings suggest that patients with conversion weakness are not simply faking their symptoms” (p. e3). Second, the authors report further functional neuroimaging studies showing that patients with conversion symptoms have relatively less activity in the right temporoparietal junction (TPJ). This, the authors conclude, “may reflect a deficit in the pathway responsible for individuals' having a sense of agency over their motor function” (p. e4). In summary, patients with conversion disorders are not faking their symptoms but rather may have no sense of agency over them, which is why we should drop the term “hysteria.” We do value the authors' conclusion that we should no longer use the semantically incorrect and discriminating term “hysteria” and speak of “functional disorders” instead. This transition, though, has been made many years ago (before functional neuroimaging provided the above cited evidence), and is already incorporated in clinical training in psychosomatics, psychiatry, psychotherapy, and adjacent disciplines. However, we would like to take this opportunity to address a more fundamental point: The authors in this opinion article implicitly assume a basic dichotomy between “fake” and “real” symptoms, between “sense of agency” and “no sense of agency” in a rather categorical way. This dichotomy is abundantly used in both scientific research and clinical practice, but, in our opinion, is highly questionable for anthropological, clinical, and ethical reasons.

## Anthropological and Clinical Aspects

The purported dichotomy of “fake” vs. “real” symptoms does not acknowledge the complexity of voluntary mental causation and perceived agency in general, and of conversion symptoms in particular. With “mental causation” we refer to the relationship between mental states (such as reasons, thoughts, and motivations) with behaviors and actions, as they are observable from a third-person perspective; “agency” is the subjectively perceived side of these relationships. Now, which mental causations are “voluntary,” associated with perceived agency, and could therefore lead to “faked” symptoms—and which are involuntary, not associated with agency, and thus qualify to generate “real” symptoms? There are good reasons to dismiss this categorical distinction and view voluntary/involuntary mental causation and agency as dimensional instead.

In general, people may have no or little awareness of the mental causes of their behavior. The dichotomy between “fake” and “real” implies a Cartesian (i.e., completely transparent) view of the mind with full awareness of the mental causes of all behaviors, feelings, and thoughts. This Cartesian view of the mind is no longer tenable, as emphasized by authors from various different traditions—including Sigmund Freud's work on the unconscious (Freud, 1955, 1957) contemporary psychodynamic thinking (Person et al., 2005) but also cognitive neuroscience theories (Cooper and Cooper, 2002; e.g., Libet, 1985; Milner et al., 1998; van Gaal et al., 2012). The role of unconscious processes is particularly important for the understanding of psychopathological symptoms: In the case of conversion disorders, which may be due to e.g., biographical conflicts and/or structural deficits (OPD-Task-Force, 2008), this lack of awareness is even among the pathogenic factors and has been variably conceptualized as repression (Freud, 1955, 1957), dissociation (Janet, 1889), alexithymia (Sifneos, 1973), or impaired mentalisation (Fonagy et al., 2002). These concepts are inherently dimensional with varying degrees of alexithymia or mentalisation capacities. Furthermore, patients may become increasingly aware of repressed contents or gain more and more control over dissociative symptoms under treatment. As time unfolds, symptoms may be maintained and reinforced—with varying degrees of awareness—by rewards (e.g., when the partner helps more with the household). This might make the conversion symptoms chronic. The simple question if these people “fake” their symptoms does not acknowledge this entire complexity. A more precise question hence could be: To which degree are people aware of the mental causes of their symptoms or have control over them?

We believe that clinical pragmatism is one major driving force behind the often assumed categorical distinctions. Clinical diagnoses are conceptualized and created in a categorical fashion—based on the fundamental dichotomy of health vs. disease—and hence need clear-cut boundaries in order to be given. This applies to the organic side (e.g., hypertension is defined by a clear blood pressure limit) as well as to the behavioral side (e.g., only fulfilling at least 5 out of 9 possible criteria for Borderline Personality Disorder justifies this diagnosis according to DSM-V). For the creation of clear guidelines in diagnosing and treating conditions, this is a very efficient approach. The problem is the inference, that the organic or behavioral “reality” is exactly mirrored by our categorical diagnostic entities. In psychosomatic disorders in general and conversion disorders in particular, “medically unexplained” symptoms have organic and mental correlates that both span well alongside a continuum. This could be exemplified by the full spectrum of ambiguity of imaging data on the organic side as well as the complex continuum between “fake” and “real” on the mental side. It is exactly this continuum that is of diagnostic relevance.

From a clinical perspective, it is not possible with current (functional) imaging technology to detect organic correlates of mental processes alongside the continuum of causes and control that is sketched above in order to help diagnosing somatoform and specifically conversion disorders. Consequently, there is currently no dimensional organic correlate of the dimensional mental process. Clinical methods on the other hand (e.g., psychodynamic or behavioral interviews, observation, questionnaires, neuropsychological testing) are relatively more advanced and have a greater chance to detect causal factors that may lie deep below the conscious level than any form of neuroimaging. This is not a principal point, though, as imaging methodology advances. Alzheimer's disease, for example, was traditionally a clinical diagnosis that is now critically supported by biological disease markers. In the area of psychosomatics and psychotherapy, an emerging literature describes brain correlates of complex phenomena such as repression (Kehyayan et al., 2013, 2018; Schmeing et al., 2013; Kessler et al., 2017), personality syndromes (Taubner et al., 2013), or treatment effects of psychodynamic therapy (Wiswede et al., 2014; Buchheim et al., 2018) as well as operant (Diers et al., 2012) or cognitive (Jensen et al., 2012) behavioral therapy. It remains to be seen how advances in imaging might improve the precision of our diagnoses in the future. For the reasons above mentioned, though, the dichotomy between “real” and “fake,” between a physical and a psychological world is as outdated as the one between free and determined actions.

## Ethical Aspects

The purported dichotomy also has highly problematic ethical implications. Since “fake” in this narrow and absolute sense has clear negative connotations, potential sanctions of fake behavior (e.g., not providing adequate therapy, interrupting the diagnostic process, or not paying insurance money) are a logical consequence. Thus, the role of a clinician becomes to try and convict a patient of lying, to detect fake symptoms in order to separate between those people to treat (“real patients”) and those to sanction (“liars”). This view can historically be traced back to the foundation of psychiatric hospitals as public detention and control institutions (Goffman, 1968; Foucault, 2013). Obviously, it conflicts with contemporary ethical standards of clinicians and with a trusting relationship between patients and clinicians. Mutual trust, transparency and the concept of informed consent before any medical decision are recent key factors in the patient-clinician relationship that are, in essence, not compatible with the absolute use of the term “fake” (Beauchamp and Childress, 2001).

## Conclusion

We criticize the dichotomy between “fake” and “real” symptoms for anthropological, clinical, and ethical reasons. Essentially, this false dichotomy in the mind of some should not be cemented by neuroimaging findings. The term “functional symptoms,” on the other hand, captures the clinical complexity more adequately. In fact, its introduction many years ago did not need neuroimaging findings to be justified. It is of crucial importance to use this term as a heuristic to describe symptoms with no (sufficient) organic correlates. It is typically well-accepted among patients and helps creating a common ground upon which patient and therapist can manage or even treat the symptoms together. Finally, we hope that this opinion article might trigger an enriched discussion concerning the adequate relevance of neuroimaging findings for complex mental processes and the way we approach such complexity.

## Author Contributions

HK, NA, MD, and SH were involved in writing and editing of the manuscript and in the discussion process before writing the manuscript.

### Conflict of Interest Statement

The authors declare that the research was conducted in the absence of any commercial or financial relationships that could be construed as a potential conflict of interest.
